# Increased levels of CRP and MCP-1 are associated with previously unknown abnormal glucose regulation in patients with acute STEMI: a cohort study

**DOI:** 10.1186/1475-2840-9-47

**Published:** 2010-09-02

**Authors:** Eva C Knudsen, Ingebjørg Seljeflot, Abdelnoor Michael, Jan Eritsland, Arild Mangschau, Carl Müller, Harald Arnesen, Geir Ø Andersen

**Affiliations:** 1 Center for Clinical Heart Research, Oslo University Hospital, Ullevål, Oslo, Norway; 2 Department of Cardiology, Oslo University Hospital, Ullevål, Oslo, Norway; 3 Center of Clinical Research, Unit of Epidemiology and Biostatistics, Oslo University Hospital, Ullevål, Oslo, Norway; 4 Center for Heart Failure Research, Oslo University Hospital, Ullevål, Oslo, Norway; 5 Faculty of Medicine, University of Oslo, Oslo, Norway; 6 Nuclear Medicine, Oslo University Hospital, Ullevål, Oslo, Norway

## Abstract

**Background:**

Inflammation plays an important role in the pathophysiology of both atherosclerosis and type 2 diabetes and some inflammatory markers may also predict the risk of developing type 2 diabetes. The aims of the present study were to assess a potential association between circulating levels of inflammatory markers and hyperglycaemia measured during an acute ST-elevation myocardial infarction (STEMI) in patients without known diabetes, and to determine whether circulating levels of inflammatory markers measured early after an acute STEMI, were associated with the presence of abnormal glucose regulation classified by an oral glucose tolerance test (OGTT) at three-month follow-up in the same cohort.

**Methods:**

Inflammatory markers were measured in fasting blood samples from 201 stable patients at a median time of 16.5 hours after a primary percutanous coronary intervention (PCI). Three months later the patients performed a standardised OGTT. The term abnormal glucose regulation was defined as the sum of the three pathological glucose categories classified according to the WHO criteria (patients with abnormal glucose regulation, n = 50).

**Results:**

No association was found between inflammatory markers and hyperglycaemia measured during the acute STEMI. However, the levels of C-reactive protein (CRP) and monocyte chemoattractant protein-1 (MCP-1) measured in-hospital were higher in patients classified three months later as having abnormal compared to normal glucose regulation (p = 0.031 and p = 0.016, respectively). High levels of CRP (≥ 75 percentiles (33.13 mg/L)) and MCP-1 (≥ 25 percentiles (190 ug/mL)) were associated with abnormal glucose regulation with an adjusted OR of 3.2 (95% CI 1.5, 6.8) and 7.6 (95% CI 1.7, 34.2), respectively.

**Conclusion:**

Elevated levels of CRP and MCP-1 measured in patients early after an acute STEMI were associated with abnormal glucose regulation classified by an OGTT at three-month follow-up. No significant associations were observed between inflammatory markers and hyperglycaemia measured during the acute STEMI.

## Background

Increased prevalence of unknown impaired glucose tolerance and type 2 diabetes has been shown in patients suffering an acute myocardial infarction (AMI) [1]. Both the short- and long-term prognoses after an AMI are worse among individuals with abnormal compared to individuals with normal glucose regulation [2]. According to guidelines, it is important to diagnose these high-risk patients with abnormal glucose regulation in order to initiate lifestyle intervention and optimal medical treatment [3].

We have recently shown that high levels of HbA1c, admission glucose, and fasting plasma glucose measured early in-hospital in patients with an acute ST-elevation myocardial infarction (STEMI) were predictive to identify patients with abnormal glucose regulation at three-month follow-up [4]. We also demonstrated poor reproducibility of an oral glucose tolerance test (OGTT) performed early after an acute STEMI compared to a new test in a stable condition three months later [4].

There is considerable evidence that inflammation plays an important role in the pathophysiology of both atherosclerosis [5] and type 2 diabetes [6] and some inflammatory markers may also predict the risk of developing type 2 diabetes [7]. Additionally, inflammation has been suggested to be the bridging link between abnormalities in glucose metabolism and atherosclerotic disorders [8] In order to elucidate a possible association between hyperglycaemia, abnormal glucose regulation and inflammation in STEMI patients without known diabetes we chose to investigate a broad panel of pro- and anti-inflammatory markers. The acute phase reactant C-reactive protein (CRP), the pro-inflammatory markers (interleukin 6 (IL-6), interleukin 8 (IL-8), monocyte chemoattractant protein-1 (MCP-1), tumor necrosis factor α (TNF-α)), soluble CD40 ligand (sCD40L), the anti-inflammatory marker adiponectin, the matrix metalloproteinase-9 (MMP-9) and its inhibitor (tissue inhibitor of metalloproteinase 1 (TIMP-1)) were investigated. We hypothesized that STEMI patients having abnormal glucose regulation would present with an increased pro-inflammatory profile.

The aims of the study were 1) to assess a potential association between circulating levels of inflammatory markers and hyperglycaemia measured during an acute STEMI in patients without known diabetes and 2) to identify a possible association between circulating levels of inflammatory markers measured acutely and abnormal glucose regulation classified by an OGTT at three-month follow-up in the same cohort.

## Methods

### Study population

The patient population has been described in detail elsewhere [4]. In brief, patients with a primary percutanous coronary intervention (PCI) treated STEMI were included if they were stable, without chest pain or nausea, age < 85 years and with serum creatinine < 200 umol/L. Patients with previously known type 2 diabetes or persistent hyperglycaemia were excluded. Patients with persistent hyperglycaemia were defined as patients with both admission plasma glucose > 11 mmol/L and a fasting capillary glucose level > 8 mmol/L before an OGTT was performed. STEMI was defined as ST-segment elevation of ≥ 2 mm in two or more contiguous chest leads, ≥ 1 mm in two or more limb leads, or new left bundle-branch block, together with typical symptoms (chest pain or discomfort > 20 min duration).

The Regional ethics committee approved the study and all patients provided written and oral informed consent.

### Laboratory methods

Admission plasma glucose concentration was analysed from blood samples taken in the catheterisation laboratory as soon as possible after PCI. Further blood samples were drawn after an overnight fast for determination of glucose, HbA1c, for routine analyses by use of conventional methods and for determination of CRP, MCP-1, TNF-α, IL-6, sCD40L, IL-8, MMP-9, TIMP-1, IL-18, and adiponectin. Serum was prepared by centrifugation within 1 hour at 2500 g for 10 min and used for all analyses, except MCP-1, which was determined in citrated plasma (0.129 mmol/L in dilution 1:10) and sCD40L determined in EDTA plasma, stored on ice and separated within 30 min by centrifugation at 4°C and 3000 g for 20 min to obtain platelet-poor plasma. All blood samples were stored at -80°C until analysis.

CRP and IL-18 were determined by enzyme-linked immunosorbent assays (DRG Instruments, Marburg/Lahn, Germany, and Medical Biological Laboratories, Naku-ku, Nagoya, Japan, respectively). MCP-1, MMP-9, TIMP-1, IL-6, IL-8, TNF-α, adiponectin and sCD40L were all measured by enzyme immunoassays from R&D Systems Europe (Abingdon, Oxon, UK).

In our laboratory, the inter-assay coefficient of variation were as follows, CRP < 5%, MCP-1 9.0%, IL-6 10.5%, IL-8 10.5%, IL-18 6.5%, TNF-α 8.5%, adiponectin 9.5%, MMP-9 7.4%, TIMP-1 4.4%, and sCD40L 9.5%.

Serum cardiac specific Troponin T (TnT) was measured by electrochemiluminescence technology for quantitative measurement (Elecsys 2010, Roche, Mannheim, Germany). The inter-assay coefficient of variation was 7%. TnT maximum was defined as the maximum value measured in each patient during the acute STEMI.

### Follow- up

Three months after the initial hospitalisation clinical examination and a standardised oral glucose tolerance test (OGTT) (75 g glucose in 200 ml water with plasma glucose measurements at 0 and 120 min) were performed [9]. The classification of glucometabolic state was based on the result of the OGTT and the patients were divided into one of the following four categories defined according to the World Health Organisation criteria [10] (glucose levels given in mmol/L):

Normal Glucose Tolerance (NGT) = OGTT (0 min) < 6.1 and OGTT (2 h) < 7.8

Impaired Fasting Glucose (IFG) = OGTT (0 min) ≥ 6.1 < 7.0 and OGTT (2 h) < 7.8

Impaired Glucose Tolerance (IGT) = OGTT (0 min) < 7.0 and OGTT (2 h) ≥ 7.8 < 11.1

Type 2 diabetes (T2DM) = OGTT (0 min) ≥ 7.0 and/or OGTT (2 h) ≥ 11.1.

The term abnormal glucose regulation was defined as the sum of IFG, IGT and T2DM.

Left ventricular ejection fraction and infarct size expressed as percent of left ventricular mass were assessed at rest at three-month follow-up by Single Photon Emission Computed Tomography imaging with technetium 99 m-tetrofosmin [11].

### Statistics

The study design was a cohort study and the outcome was defined as abnormal glucose regulation. We hypothesised an association between inflammatory variables and the state of glucose regulation. Continuous variables were categorised into quartiles. A linear trend analysis across the quartiles of an inflammatory marker identified the cut off point used. The Mantel-Haenszel method was used to highlight potential effect modification by the Breslow-Day test of heterogeneity and to quantify potential confounders [12]. Additional information is available online [Additional file 1: Supplemental Table S1]. The following risk factors were analysed as potential confounders; gender, age, current smoking, treated hypertension, TnT maximum, body mass index, cholesterol, triglycerides, CRP and MCP-1. A logistic regression model, including a backward elimination procedure was performed to adjust for the confounders.

Continuous variables are presented as median and 25, 75 percentiles and categorical variables as proportions. The correlations between the investigated variables were assessed by use of Spearman's rho and adjustments were performed using a multivariate analysis with logarithmically transformed data. The STROBE guidelines were followed [13]. A value of p < 0.05 was considered statistically significant. All analyses were performed using Epi-info software, 2005, version 3.3.2, except Spearman correlation coefficient analyses and multiple regressions, which were made by use of SPSS software, 2006, version 15.0 (SPSS, Chicago, L).

## Results

### Baseline characteristics

Two hundred and one patients with a primary PCI treated STEMI were enrolled in the study [4]. Baseline characteristics are shown in Table 1. Notably, BMI was 26 kg/m^2^, 82% were men and 61% had single vessel disease. Fasting blood samples were drawn at a median time of 20 h and 20 min after the occurrence of symptoms and 16 h and 35 min after the primary PCI.

**Table 1:** Baseline characteristics of the total population (n = 201).

	Patients
Age (years)	58 (51, 67)
Male	185 (82.6%)
Previous disorder:	
Myocardial infarction	16 (7.1%)
Angina pectoris	7 (3.1%)
Hypertension (treated)	58 (25.9%)
Hyperlipidaemia (treated)	20 (8.9%)
Status at baseline	
Current smoker	109 (48.7%)
TnT maximum (ug/L)	4.70 (2.45, 8.92)
BMI (kg/m2)	26 (24.4, 28.7)
Waist circumference (cm)	100 (94, 107)
Stent in culprit lesion	215 (96.0%)
Gp IIb/IIIa antagonist treated	79 (35.3%)
Single -coronary vessel disease	139 (62.1%)
Double-coronary vessel disease	64 (28.6%)
Triple-coronary vessel disease	21 (9.4%)
Time from symptoms to balloon (min)	219 (140, 378)
Medication at three months (n = 201)	
Aspirin	200 (99.5%)
Clopidogrel	189 (94%)
β-blockers	188 (93.5%)
Lipid lowering agents	194 (96.5%)
Angiotensin converting enzyme-inhibitors	69 (34.3%)
Angiotensin II-receptor blockers	25 (12.4%)
Glucose lowering medication	0
LVEF^a ^(%)	64 (56, 70)
Infarct size^a^, % of left ventricular mass	14.0 (0.0, 29.0)

Data are presented as median (25, 75 percentiles) values or proportions.

BMI: body mass index, LVEF: left ventricular ejection fraction, TnT maximum: serum cardiac specific Troponin T maximum.^a^LVEF and infarct size were measured at three-month follow-up by SPECT.

The plasma glucose concentration at admission was 6.9 (6.0, 7.8) mmol/L, the fasting plasma glucose 5.3 mmol/L (4.9, 5.9) and HbA1c 5.5% (5.3, 5.8). As previously reported, patients defined with abnormal glucose regulation were older, there were more women, and they had significantly higher levels of HbA1c, admission plasma glucose, and fasting plasma glucose measured in-hospital, compared to patients with normal glucose regulation [4].

### Follow-up characteristics

All patients were reached to follow up. However, one patient did not perform the OGTT because the level of fasting glucose measured was above 7 mmol/L. At three-month follow-up the levels of fasting plasma glucose and HbA1c were 5.2 mmol/L (4.8, 5.6) and 5.6% (5.4, 5.8), respectively. The prevalence of abnormal glucose regulation based on the OGTT classification was 25% (n = 50). The prevalence of impaired fasting glucose, impaired glucose tolerance, and type 2 diabetes were 5.5% (n = 11), 14.4% (n = 29) and 5% (n = 10), respectively. The medications for secondary prevention recorded were according to current guidelines, i.e. 99.5% were on aspirin and 96.5% were on lipid lowering agents (Table 1).

### Inflammatory markers and hyperglycaemia

As can be seen from Table 2, there were no associations between admission glucose and the inflammatory markers measured whereas significant correlations were observed between fasting glucose and IL-6 (p = 0.004) and adiponectin (p = 0.038). However, after adjustment for age and TnT maximum measured in-hospital, no significant associations were found (p = 0.14 and p = 0.095, respectively).

**Table 2:** Correlations between the inflammatory markers and admission plasma glucose (APG) and fasting plasma glucose (FPG), all measured acutely in-hospital.

Variables	APG		FPG	
	r_s_	p	r_s_	p
CRP mg/L	0.04	NS	0.13	NS
MCP-1 pg/mL	0.05	NS	-0.11	NS
TNF-α pg/mL	0.06	NS	-0.02	NS
IL-6 pg/mL	-0.04	NS	0.20	0.004
sCD40L pg/mL	-0.06	NS	-0.05	NS
IL-8 pg/mL	-0.12	NS	-0.12	NS
TIMP-1 ng/mL	0.06	NS	0.03	NS
MMP-9 ng/mL	0.13	NS	0.13	NS
IL-18 pg/mL	-0.03	NS	-0.04	NS
Adiponectin ng/mL	-0.03	NS	0.15	0.04

r_s _indicate Spearman correlation coefficient.

Abbreviations: see main text.

NS: non-significant.

### Inflammatory markers and abnormal glucose regulation

Elevated levels of CRP (p = 0.031) and MCP-1 (p = 0.016) measured in-hospital were found in patients classified with abnormal glucose regulation at three-month follow-up (Table 3). There was no difference between the two groups according to the other inflammatory markers measured (Table 3). Furthermore, only a weak correlation between MCP-1 and CRP was found (r = -0.14, p = 0.049).

**Table 3:** Levels of inflammatory markers measured in-hospital related to normal (NGR) and abnormal glucose regulation (AGR) categorised by an OGTT after three months.

Variables	NGR (n = 151)	AGR (n = 50)	P
CRP mg/L	10.99 (5.95, 30.0)	20.91(8.40, 41.98)	0.031
MCP-1 pg/mL	218 (185, 268)	241 (205, 301)	0.016
TNF-α pg/mL	1.51 (1.25, 2.06)	1.58 (1.26, 2.06)	0.996
IL-6 pg/mL	17.16 (11.22, 28.04)	20.88 (13.76, 31.00)	0.131
CD40L pg/mL	63.3 (44.5, 97.4)	64.6 (43.3, 88.8)	0.599
IL-8 pg/mL	15.1 (13.6, 17.8)	15.4 (13.2, 19.4)	0.432
TIMP-1 ng/mL	190 (162, 217)	199 (163, 220)	0.911
MMP-9 ng/mL	502 (341, 664)	539 (414, 748)	0.254
IL-18 pg/mL	269 (203, 333)	288 (239, 359)	0.096
Adiponectin ng/mL	4794 (3006, 7759)	4385 (2806, 7075)	0.474

Median values (25, 75 percentiles) are given.

Abbreviations: see main text.

P-values refer to difference between groups.

**Figure 1:**
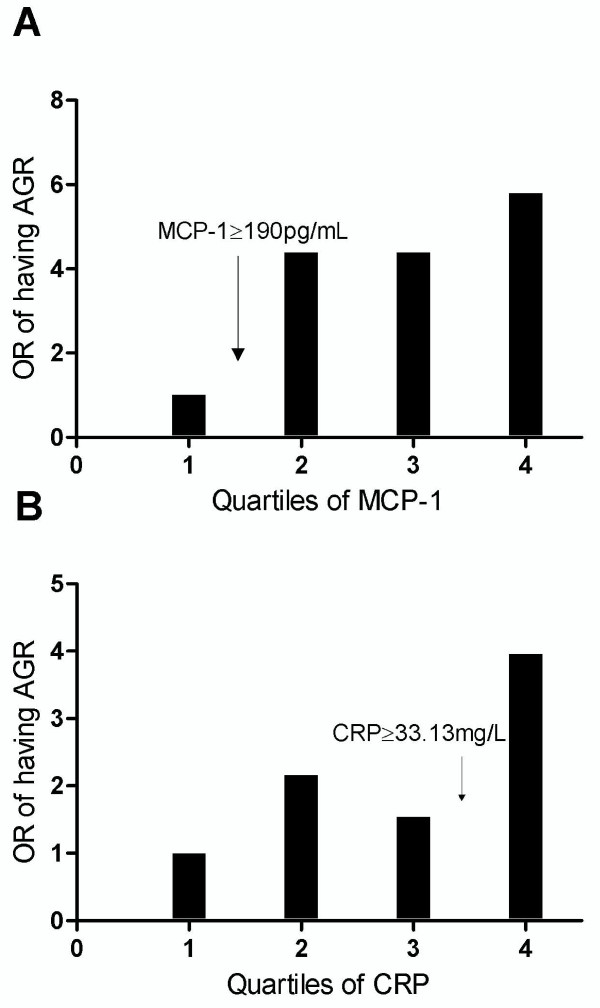
**Odds ratios of having abnormal glucose regulation classified three months after an acute STEMI by quartiles of MCP-1 (pg/mL, panel A) and CRP (mg/L, panel B) measured in-hospital**. P for trend, (A, p = 0.005) and (B, p = 0.016).

When dividing the MCP-1 and CRP levels into quartiles, there were significant trends for the presence of abnormal glucose regulation with increased levels of both biomarkers (p = 0.005 and p = 0.016, respectively), identifying a threshold for MCP-1 at the 25 percentile (190 ug/mL) and for CRP at the 75 percentile (33.13 mg/L) (Figure 1A and 1B). In univariate analyses, high levels of CRP (≥ 75 percentile) and MCP-1 (≥ 25 percentile) in-hospital were associated with abnormal glucose regulation classified at three-month (p = 0.007 and p = 0.004, respectively) (Table 4). CRP remained associated with abnormal glucose regulation after adjustment for potential confounders with an OR 3.24 (95% CI 1.54, 6.83) (Table 4). Triglycerides were shown to modify the association between MCP-1 and abnormal glucose regulation (Table A online). Consequently, the patients were divided into two strata with high and low triglyceride levels. High levels of MCP-1 were independently associated with abnormal glucose regulation in 150 patients with triglycerides below the 75th percentile (1.8 mmol/L) with an OR 7.56 (95% CI 1.67, 34.18).

**Table 4:** Crude and adjusted OR of the association between high levels of CRP and MCP-1 measured in-hospital and abnormal glucose regulation defined by an OGTT three months later using logistic regression analyses.

	AGR		AGR		AGR TG < 1.8 mmol/l (n = 150)		AGR TG ≥ 1.8 mmol/l (n = 49)	
	**OR (crude)** **(95% CI)**	**P**	**OR (adjusted)** **(95% CI)**	**P**	**OR (adjusted)** **(95% CI)**	**P**	**OR (adjusted)** **(95% CI)**	**P**

CRP^a^ (≥ 33.13 mg/L)	2.58 (1.29, 5.14)	0.007	3.24 (1.54, 6.83)	0.002				
MCP-1^b^ (≥ 190 pg/mL)	4.82 (1.64, 14.20)	0.004			7.56 (1.67, 34.18)	0.009	1.81 (0.16,19.87)	0.626

TG: triglycerides. AGR: abnormal glucose regulation. Abbreviations: see main text.

^a ^Adjusted for identified confounders (gender and MCP-1).

^b ^Stratified for triglycerides as an effect modifier and adjusted for identified confounders (age, gender, treated hypertension, TnT maximum and CRP).

## Discussion

The main results of the present study were that elevated levels of CRP and MCP-1 measured in patients early after an acute STEMI were associated with abnormal glucose regulation defined at three-month follow-up. Additionally, during the acute STEMI, there were weak, non-significant associations between fasting glucose and IL-6 and adiponectin, while there was no association between admission glucose and the inflammatory markers measured.

### Inflammatory markers and hyperglycaemia

During experimental conditions, induction of hyperglycaemia in humans has been shown to increase circulating levels of cytokines and the effect was more pronounced in subjects with impaired glucose tolerance suggesting a causal role of hyperglycaemia in the activation of the inflammation in diabetes [14]. Furthermore, hyperglycaemia at admission has been reported to be associated with increased risk of in-hospital mortality and poor long-term outcome [15-17]. There are, however, no guidelines defining the level of pathological hyperglycaemia at admission with an acute myocardial infarction [17].

It has been shown that patients with a primary PCI treated STEMI generate a marked, short-term increase in circulating levels of inflammatory markers [18] and higher levels of CRP and IL-6 in patients with acute myocardial infarction and diabetes compared to patients without diabetes have also been reported [19]. In the present study we could, however, not reveal any association between the acute hyperglycaemia and the inflammatory responses. The relatively few patients with hyperglycaemia at admission may partly explain these negative results. In addition, we may have missed the peak levels of the measured inflammatory markers by the delayed sampling time point. The association found between fasting glucose and IL-6 and adiponectin probably reflect a relation to infarct size because it was no longer significant after adjustment for TnT maximum.

### Associations between inflammatory markers and abnormal glucose regulation

We have recently shown that in patients with a primary PCI treated STEMI, a very early OGTT should probably not be recommended because of lack of reproducibility [4]. However, high levels of admission glucose, fasting glucose, and HbA1c in these patients were shown to be associated with abnormal glucose regulation defined at three-month follow-up [4]. In the present study, searching for novel biomarkers that may associate with abnormal glucose regulation, we found that high levels of CRP and MCP-1 measured early after an acute STEMI were associated with abnormal glucose regulation diagnosed in a stable cituation. The associations found between high levels of CRP and MCP-1 measured acutely and abnormal glucose regulation defined three months later might be influenced by the acute STEMI, which are known to release several inflammatory mediators from the necrotic myocardium into the circulation [20].

Our findings may be interpreted along with previous results showing higher levels of CRP in patients with abnormal compared to those with normal glucose regulation [21] thus the high levels of CRP measured acutely may reflect a low-grade systemic inflammation in glucose intolerant patients.

MCP-1 levels have been reported to be elevated in patients with an acute myocardial infarction [22] and poor glycemic control has been suggested to induce high levels of MCP-1 in diabetic patients [23]. Furthermore, increased monocyte recruitment into the subendothelial space has been shown in patients with diabetic angiopathy and MCP-1 seems to play a key role in this process [24]. In our study, the association between high levels of MCP-1 and abnormal glucose regulation may indicate that patients with an abnormal glucometabolic status do present with high levels of MCP-1 before the acute STEMI or it might be suggested that patients with abnormal glucose regulation respond with higher levels of MCP-1 in the acute phase, compared to those with normal glucose regulation.

MCP-1 levels have been shown to correlate with triglyceride levels in post-menopausal women [25] and in patients with diabetes [23]. In our study the association between MCP-1 and abnormal glucose regulation was present in the majority of the patients, but not in patients with triglycerides above 1.8 mmol/L, suggesting that in these patients the high levels of triglycerides may mask the association between MCP-1 and glucose regulation.

CRP and MCP-1 were associated with abnormal glucose regulation independently of each other, indicating that these markers probably are involved in different pathological processes associated with type 2-diabetes.

MCP-1 and IL-8 are functionally related, potent chemoattractants both being shown to be involved in the atherosclerotic process [26]. It has previously been reported that fasting levels of IL-8 correlated with BMI both in subjects with normal and impaired glucose tolerance [27]. In obese subjects without coronary heart disease, the post-load levels of IL-8 increased after an OGTT in subjects with impaired glucose tolerance compared to normoglycaemic weight-matched individuals [27] However, we did not found any association between IL-8 and abnormal glucose regulation.

Elevated levels of IL-18 have been associated with an increased risk of developing type 2-diabetes [28]. Also high levels of MMP-9 and TIMP-2 have been found in patients with an acute coronary syndrome and type 2 diabetes, probably reflecting an abnormal extracellular matrix metabolism in these patients [29]. On the contrary, circulating levels of adiponectin, which is an antiatherogenic, anti-inflammatory and insulin-sensitizing adipokine, have been shown to be lower in patients with type 2 diabetes and macro vascular disease than those without [30]. We found, however, no associations between abnormal glucose regulation and the levels of IL-18, MMP-9, TIMP-1, and adiponectin. This may be explained by the fact that only a small number of our patients were classified as having type 2-diabetes.

Almost all the patients in the present study were treated according to guidelines for secondary prevention with medication, which included lipid lowering agents and anti-platelet treatment. However, glucose lowering medication, which could have had a confounding effect on the glucometabolic classification, was not introduced.

### Study limitations

Unstable patients with cardiogenic shock, renal failure, ongoing chest pain, nausea and persistent hyperglycaemia were excluded from the study, possibly making a selection bias towards more glucometabolically normal patients.

## Conclusion

Elevated levels of CRP and MCP-1 measured early after a primary PCI treated STEMI in patients without previously known diabetes were associated with abnormal glucose regulation classified by an OGTT at three-month follow-up indicating an important role of low grade inflammation in glucose regulation. There was however, non-significant association between inflammatory markers and hyperglycaemia during the acute STEMI in the same cohort

## Competing interests

The authors declare that they have no competing interests.

## Authors' contributions

ECK performed the statistical analysis of the data presented and drafted the manuscript.

MA made substantial contribution with statistical analysis. GØA contributed with the conception and design of the study. ECK, IS, MA, JE, AM, HA and GØA participated in the study design and interpretation and revised the manuscript critically for important intellectual content. All authors have read and approved the final manuscript.

## Supplementary Material

Additional file 1 **Stratified analysis on major potential confounders using the Mantel-Haenszel method**. Table S1 shows identified effect modifiers and potential confounders in the associations between CRP and AGR, and MCP-1 and AGR by use of the Mantel-Haenszel method. Abbreviations: see main text. Click here for file
