# Supplementary material for: Increased levels of CRP and MCP-1 are associated with previously unknown abnormal glucose regulation in patients with acute STEMI: a cohort study
Source: Cardiovasc Diabetol. 2010 Sep 2;9:47. doi: 10.1186/1475-2840-9-47 (PMC2940874; doi:10.1186/1475-2840-9-47)
Supplement: Additional file 1 — Stratified analysis on major potential confounders using the Mantel-Haenszel method. Table S1 shows identified effect modifiers and potential confounders in the associations between CRP and AGR, and MCP-1 and AGR by use of the Mantel-Haenszel method. Abbreviations: see main text. [file 1475-2840-9-47-S1.DOC]

Table S1 Stratified analysis on major potential confounders using the Mantel-Haenszel method.

|  | AGR- | AGR+ | OR  95%CI | ORMH  95%CI | Breslow &Day  Heterogeneity test |
| --- | --- | --- | --- | --- | --- |
| MCP-1  <190 pg/ml  ≥190  Gender  Male  Female  Crude effect | CRP≥33.13  no yes  33 12  86 19  100 29  20 2  120 31 | CRP≥33.13  no yes  1 3  28 17  22 14  8 6  30 20 | 8.25 (0.78, 87.17)  2.75 (1.26, 6.00)  2.19 (1.00, 4.82)  7.50 (1.24, 45.29)  2.58 (1.29, 5.15) | 3.10 (1.49, 6.46)  2.74 (1.35, 5.55) | P  0.386  0.220 |
| CRP  <33.13 mg/l  ≥33.13  Age  <68 years  ≥68  Gender  Male  Female  ScTnT  <8.81ug/l  ≥8.81  Triglycerides  <1.8 mmol/l  ≥1.8  Treated HT  No  Yes  Crude effect | MCP-1≥190  No yes  33 86  12 19  36 83  9 22  38 90  7 15  34 82  11 23  39 75  6 30  39 73  6 32  45 105 | HighMCP-1≥190  No yes  1 28  3 17  3 26  1 19  2 34  2 11  4 28  0 17  2 34  2 11  2 30  2 15  4 45 | 10.74 (1.40, 82.19)  3.58 (0.86, 14.87)  3.76 (1.07, 13.22)  7.77 (0.90, 67.08)  7.18 (1.64, 31.40)  2.57 (0.44, 14.82)  2.90 (0.95, 8.91)  17.13 (0.94, 311)  8.84 (2.02, 38.74)  1.10 (0.19, 6.29)  8.01 (1.82, 35.32)  1.41 (0.25, 7.80)  4.82 (1.64, 14.21) | 6.03 (1.90, 19.10)  4.58 (1.55, 13.51)  5.16 (1.70, 15.63)  4.20 (1.51, 11.62)  *  4.48 (1.53, 13.13) | 0.386  0.568  0.379  0.264  0.074  0.132 |
|  |  |  |  |  |  |

*Cannot be calculated because triglycerides are an effect modifier of MCP-1 on abnormal glucose regulation (AGR).

In the 2x2 table with zero cells +0.5 have been added to the 4 cells to be able to calculate the stratum specific OR and the Mantel-Haenszel summary OR.

The confounding effect is quantified using the formula ORMH-ORcrude

ORcrude
